# Assessing the association between multiple indicators of inflammation and sleep disorders in young and middle-aged women: insights from traditional and machine learning approaches

**DOI:** 10.1186/s40001-025-03203-0

**Published:** 2025-10-15

**Authors:** Yi Tang, Kangrui Zhang, Xin Tang, Yueyu Zhang, Jiaxuan Li, Xinhui Jia, Xun He, Xinyi Chen, Jie Hu, Zhinan Ye, Juncang Wu

**Affiliations:** 1 Department of Neurology, Anhui Medical University, Hefei, 230032 China; 2 Department of Neurology, The Second People’s Hospital of Hefei, Hefei, 230011 China; 3 Department of Orthopaedic Surgery, Jiangxi Medical College, Nanchang University, Nanchang, 330031 China; 4 Department of Neurology, School of Medicine, Taizhou Municipal Hospital (Taizhou University Affiliated Municipal Hospital), Taizhou University, Taizhou, China

**Keywords:** Alpha-1-acid glycoprotein, Inflammatory markers, Sleep disorder, NHANES

## Abstract

**Background:**

Interactions between inflammation and sleep disorders are increasingly recognized; however, limited research comprehensively evaluates the association between multiple inflammatory indicators and sleep disorders.

**Methods:**

This cross-sectional study utilized data from the National Health and Nutrition Examination Survey (NHANES, 2015–2020) involving 2,342 participants. Machine learning algorithms were employed to identify inflammatory indicators with potential predictive value for sleep disorders, followed by Shapley value analysis to quantify their contributions. Weighted logistic regression and restricted cubic spline models were applied to examine associations between key inflammatory markers and sleep disorders. Mediation analysis was conducted to assess the role of depression in these relationships. Receiver operating characteristic (ROC) curves were generated to compare the predictive performance of individual inflammatory markers. Sensitivity analyses using E-values were performed to evaluate the robustness of findings against unmeasured confounding.

**Results:**

Alpha-1-acid glycoprotein, C-reactive protein, and Naples Prognosis Score all showed significant positive correlations with sleep disorders. Among these, AGP and CRP contributed most significantly to the model (Shap value≈0.23). Furthermore, mediation analysis indicated that depression mediated 15.1% of the total effect of AGP on sleep disorders.

**Conclusions:**

The study confirms a significant positive association between serum AGP levels and sleep disorders. Among the inflammatory markers evaluated, AGP exhibited the strongest correlation, underscoring its potential clinical relevance in the pathophysiology of sleep disturbances.

**Supplementary Information:**

The online version contains supplementary material available at 10.1186/s40001-025-03203-0.

## Background

A growing body of evidence from prospective cohort studies indicates that sleep disorders can promote enhanced inflammatory responses. Established inflammatory markers such as the Systemic Inflammation Index (SII), C-reactive protein (CRP), and neutrophil-to-lymphocyte ratio (NLR) have been extensively demonstrated to correlate significantly with sleep disturbances[1–3]. Nevertheless, the association between sleep disorders and several novel inflammatory parameters remains underexplored. These include high-density lipoprotein (HDL)-related inflammation indices, alpha-1-acid glycoprotein (AGP), the Naples Prognostic Score (NPS), and the neutrophil percentage-to-albumin ratio (NPAR).

This study categorizes these biomarkers into three groups based on their pathophysiological properties. The first is composite inflammatory indices: the HDL-related inflammatory indices, NPAR, and SII, which belong to a new class of inflammatory markers and are likely to reflect the inflammatory and immune status of the body better than individual blood cells or lipid markers[4]. The second group is acute-phase response proteins, AGP and CRP. There have been numerous studies confirming that sleep disorders are associated with CRP and IL-6[3]. AGP, as an acute-phase plasma protein, is synthesized through the co-regulation of IL-1β, TNF-α, and IL-6 [5, 6]. It exhibits immunomodulating activity, regulating acute inflammatory responses, and possesses anti-inflammatory and antibacterial effects[7]. In addition, this protein can bind to various molecules such as alkaline drugs and steroid hormones, exhibiting characteristics with potential physiological significance[6]. Unlike other inflammatory markers, it remains stable or decreases under estrogen exposure[8]. It has been demonstrated that AGP levels are elevated in the urine of children with obstructive sleep apnea (OSA) [8, 9], indicating that it is involved in the activation of inflammatory pathways. Furthermore, proteomics-based ROC analysis validates the diagnostic value of AGP[9]. The third is a comprehensive prognostic scoring system: the Naples Prognostic Score (NPS). It was initially used for prognostic assessment of colorectal cancer[10], but is now also used for asthma and cardiovascular disease because it reflects the inflammatory and nutritional status of the patient[11, 12]. As a complex scoring system, it may also have some predictive power for sleep disorders.

Sleep disorders, prevalent clinical conditions, impair physical and mental health by inducing oxidative stress imbalance and proinflammatory states, underscoring the urgent need for early warning biomarkers [13–15].

Utilizing data from the National Health and Nutrition Examination Survey (NHANES, 2015–2020), this study aims to evaluate the association and predictive capacity of these novel inflammatory biomarkers for sleep disorders, integrating machine learning approaches with traditional statistical methods to enhance the robustness and interpretability of our findings.

## Methods

The investigation was organized into a three-phase methodological framework. In the first phase, machine learning models were used to systematically identify screening from multiple inflammatory indicators with potential relevance to sleep disorders. Subsequently, the second phase was quantified using multiple regression analysis and mediation modeling while controlling for confounding factors such as demographics, lifestyle, and depression. In the third stage, ROC curves were used to compare the predictive power of individual inflammatory indicators with sleep disorders.

## Study population

**Fig. 1:**
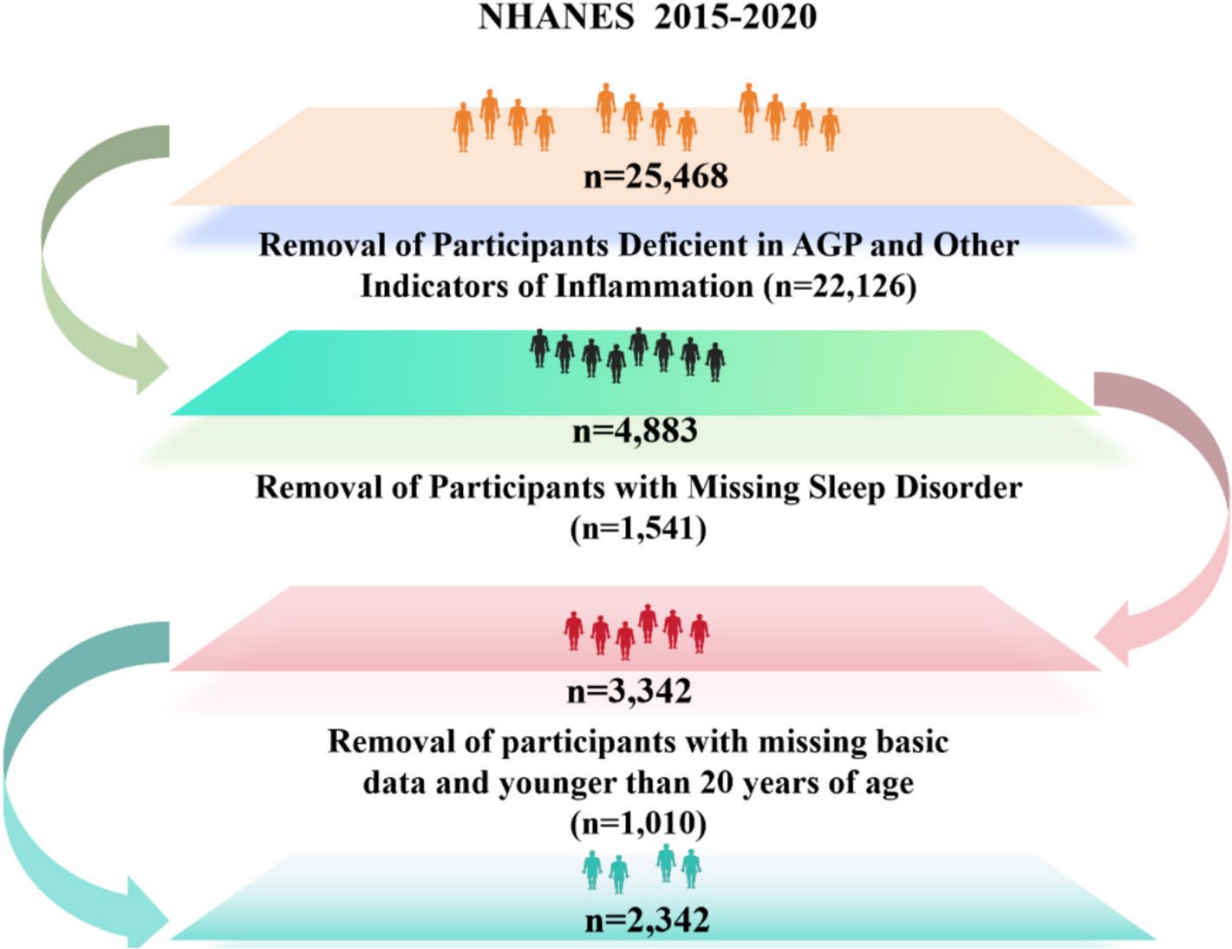
Flowchart of participant selection. Missing data in covariates included: 16 individuals with missing data on diabetes, hypertension, educational attainment, and smoking/alcohol consumption; 325 individuals with missing data on OSA diagnostic criteria

The CDC conducted a nationally representative survey of health and nutritional screening called NHANES. The National Center for Health Statistics (NCHS) Research Ethics Review Board approved the study procedures[16]. At the time of participation in this study, all participants signed a paper consent form. The scope of our current study was 6 years of data from March 2015 through December 2020. We excluded 20,585 participants with missing AGP and inflammatory cell, albumin, and HDL data, deleted 1541 participants with missing sleep outcomes, excluded 559 participants younger than 20 years of age, and deleted 438 participants with missing covariates. A total of 2342 participants were included in this study (Fig. 1). Additionally, males were excluded from this study because the NHANES database lacked laboratory data for AGP in males.

## Results and exposure factors

Sleep disorder classification employed a dual-measure paradigm combining objectively quantified duration deviations and self-perceived sleep impairment. Participants self-reported habitual nocturnal sleep patterns using the validated question: 'How many hours do you typically sleep during workweek nights?' Responses were categorized as shortened (< 7 h/night), normative (7-9 h/night), or prolonged (> 9 h/night) based on NIH Sleep Research Society criteria[17]. Subjective sleep distress was identified through affirmative responses to the clinician-administered item: 'Have you ever discussed persistent sleep difficulties with a healthcare provider?' [13, 18]. Following the ICSD-3 diagnostic algorithms [19], individuals with either non-normative sleep duration or clinically acknowledged sleep concerns were designated as having sleep disorders. This approach is consistent with emerging evidence that emphasizes the phenomenological nature of sleep disorders. As demonstrated by Buysse’s neurophenomenological studies, subjective perception plays a critical role in sleep pathology [20], underscoring the importance of integrating both objective and subjective measures in sleep disorder assessment.

The exposure factor we used was alpha-1-acid glycoprotein-serum (SSAGP) from laboratory data in the NHANES database. In our analyses, we considered AGP levels as continuous variables and grouped participants according to AGP quartiles for further analysis. Specific measurement details regarding AGP and the calculation method for inflammatory composite indices are provided.

### Covariables

In this study, the following covariates were included to improve the stability of the relationship between exposure factors and outcomes: age, race, family PIR, body mass index, education, hypertension, and diabetes mellitus, referring to the published literature on the subject [21–23]. Mexican American, Other Hispanic, Non-Hispanic White, Non-Hispanic Black, and Other were the five categories into which we divided racial categories. The categories used to describe achievement in education were below secondary school level, high school students in grades 9 to 11, high school graduates, holders of an Associate’s degree, and holders of a postgraduate degree [24]. According to the definition criteria for research variables, participants’ relevant behavioral indicators are defined as follows: 1. smoking status: categorized based on whether participants’ lifetime cumulative smoking volume reaches 100 cigarettes. 2. Drinking status: classified as drinkers if daily alcohol intake reaches or exceeds 4/5 standard drinks. 3. Physical activity level: involves sustained moderate-intensity activity lasting at least 10 min.

The PHQ-9 scale has become a key self-report instrument in primary health care and research by enabling rapid assessment of depressive symptoms through nine items. Its factor model is stable and has good psychometric properties [25]. Higher scores on a total score of 0–27 correspond to more severe depressive states. When using a score of 10 as a diagnostic threshold, the sensitivity and specificity for recognizing major depression were 88% [26].

According to the diagnostic criteria for OSA (obstructive sleep apnea) established in earlier studies, an individual may be diagnosed with OSA if they meet any one of the following conditions in the NHANES questionnaire: daytime sleepiness: despite sleeping ≥ 7 h per night, experiencing 16–30 episodes of unrelieved drowsiness per month; nocturnal breathing abnormalities: occurrence of snoring, interrupted snoring, or apnea ≥ 3 nights per week; frequent snoring: Noticeable snoring ≥ 3 nights per week [27].

### Statistical analysis

All analyses accounted for NHANES’s multistage sampling design through probability weighting, stratification, and cluster adjustments to ensure nationally representative estimates. Weighted means and frequency distributions are reported unless specified, per NHANES analytical protocols.

An initial assessment of normality via the Kolmogorov–Smirnov test revealed a non-normal distribution of data. For non-normally distributed continuous variables, the median and interquartile range were employed for data description, with the Kruskal–Wallis H test utilized for intergroup comparisons. Categorical data were presented using proportions, with the Chi-square test applied for categorical variables [28]. Our statistical evaluations were performed using R (http://www.r-project.org) and EmpowerStats (http://www.empowerstats.com), and statistical significance was indicated as significant only when *P* < *0.05*.

To identify variables associated with sleep disorders, we performed an initial screening using Lasso regression and resilient network regression models, retaining covariates screened by both algorithms. Subsequently, covariates were investigated using a variance inflation factor (VIF) approach, and variables with a VIF greater than 10 were excluded from subsequent machine learning (ML) modeling. To mitigate the category imbalance between the comorbidity and non-comorbidity groups, a Smoot sampling technique was used. 11 different machine learning models were subsequently developed: Decision Tree (DT), Adaptive Boosting (AdaBoost), Random Forest (RF), ​Naive Bayes (NB), ​Categorical Boosting (CatBoost), ​eXtreme Gradient Boosting (XGB), ​Light Gradient Boosting Machine (LGBM), ​Gradient Boosting Decision Tree (GBDT), ​Support Vector Machine (SVM), ​MultiLayer Perceptron (MLP), ​Logistic Regression (LR). The area under the curve (AUC) was the primary metric used to evaluate the models. Other performance metrics include accuracy, prevalence, sensitivity, F1-score, Matthews correlation coefficient (MCC), precision (positive predictive value), false negative rate (FNR), and false positive rate (FPR). The stability of the best model was then assessed using fivefold cross-validation. A Shapley Additive exPlanation (SHAP) analysis was then performed on the most validated models to assess the significance of features and to identify indicators of inflammation that were significantly associated with sleep disorders.

We categorized the quartiles of AGP, PHR, and CRP into Q1, Q2, Q3, and Q4. NPS was divided into 3 groups, with a score of 0 as Q1, a score of 1–2 as Q2, and a score of 3–4 as Q3. This approach evaluated the relationships between serum AGP, PHR, CRP, and NPS and sleep disorders in more detail. We also applied multivariate logistic regression to explore the relationship between serum AGP as a continuous variable and AGP as a subgroup with sleep disorders, respectively. Finally, we performed a mediation analysis using the coefficient product method to calculate the indirect effect of depression-mediated AGP on sleep disorders compared with the total effect of AGP on sleep disorders [29].

In considering confounding variables, we referred to several risk factors documented in the literature that can lead to sleep disorders [30]. In Model 1, we did not adjust for covariates and included only the association between serum AGP and sleep disorder. In Model 2, we adjust for demographic characteristics such as age, educational attainment, race, and family income-to-poverty ratio (PIR). In Model 3, we adjusted for all covariates, including age, race, family PIR, education, BMI, alcohol use, smoking, moderate-intensity activity, depression, diabetes, and hypertension.

In subgroup analyses, we used interaction tests to explore whether the association between serum AGP, PHR, CRP, and NPS and sleep disorder was significant in the subgroups of family PIR, race, education, age, and BMI. We trichotomized age according to the number of participants. Family PIR values were categorized as < 1.3, 1.3–3.5, and ≥ 3.5. The BMI was divided into 25 < , 25–29.9, and ≥ 30 kg/m^2^ to represent the various demographics, including normal weight, overweight, and obese. OSA is classified as yes or no.

To quantify the predictive ability of inflammatory markers such as AGP, CRP, PHR, NPS, SII, and NPAR for sleep disorders, their diagnostic value was analyzed using reliability operating characteristic curves (ROCs), and ROC curve integrals (AUCs) were calculated using the C-statistic.

Additionally, we explored the effect of possible unmeasured confounders between AGP and sleep disorders by calculating E-values [31]. The E-value quantifies the magnitude of unmeasured confounders that may be required to counteract the observed association between AGP and sleep disorders.

## Results

### Baseline characteristics

The study included 2,342 female participants, ranging in age from 20 to 49. The mean serum AGP concentration of the sample was 0.791 g/L, and the odds of sleep disorder prevalence were 49.39%. Their mean age was 34.503 years. The highest percentage of non-Hispanic Whites was 33.13%; the second highest was non-Hispanic Blacks at 22.2%, other races at 17.46%, Mexican-Americans at 16.61%, and other Hispanics at 10.59%. Table 1 presents the characteristics of participants stratified by AGP quartiles. The family poverty–income ratio (PIR) demonstrated lower values in the highest AGP quartile (Q4) compared with the lowest quartile (Q1). Conversely, significantly elevated body mass index (BMI), C-reactive protein (CRP) levels, and higher diabetes prevalence were observed in Q4 relative to Q1.

**Table 1:** Baseline characteristics of weighted samples by serum AGP quartile classification

Serum AGP(g/L)					
	Q1	Q2	Q3	Q4	P-value
AGE	33.213 ± 8.563	34.147 ± 8.477	35.693 ± 8.783	34.939 ± 8.508	< 0.001
BMI	24.643 ± 5.313	28.137 ± 6.700	32.049 ± 7.749	35.561 ± 8.902	< 0.001
Family PIR	2.808 ± 1.611	2.594 ± 1.564	2.288 ± 1.528	2.160 ± 1.495	< 0.001
Lymphocyte count, × 103 cells/uL	2.353 ± 0.755	2.311 ± 0.728	2.284 ± 0.718	2.340 ± 0.705	0.362
Neutrophil count, × 103 cells/uL	4.570 ± 1.959	4.520 ± 1.762	4.423 ± 1.663	4.592 ± 1.869	0.382
Platelet count, × 103 cells/uL	265.159 ± 62.689	270.719 ± 64.329	266.300 ± 66.737	272.721 ± 71.613	0.163
Neutrophil percentage (%)	57.067 ± 9.843	57.615 ± 8.810	57.240 ± 8.995	59.907 ± 8.419	< 0.001
Monocyte, × 10^3^ cells/uL	0.509 ± 0.161	0.535 ± 0.174	0.539 ± 0.168	0.594 ± 0.191	< 0.001
Albumin(g/dL)	4.068 ± 0.370	4.074 ± 0.364	4.059 ± 0.396	4.072 ± 0.390	0.907
HDL (mg/dL)	66.565 ± 16.142	58.953 ± 16.375	53.879 ± 16.170	49.242 ± 14.651	< 0.001
Total cholesterol (mg/dL)	181.841 ± 39.154	179.799 ± 38.002	183.951 ± 38.711	181.056 ± 34.112	0.284
CRP	4.317 ± 5.985	4.360 ± 6.160	4.966 ± 8.770	5.068 ± 8.219	0.172
NLR	2.058 ± 0.971	2.078 ± 0.933	2.082 ± 0.979	2.106 ± 1.188	0.885
LMR	5.089 ± 2.357	4.737 ± 2.078	4.641 ± 2.131	4.329 ± 1.889	< 0.001
SII	546.525 ± 295.552	564.186 ± 294.068	550.454 ± 285.318	577.630 ± 353.500	0.294
NPAR	1414.281 ± 276.301	1427.319 ± 266.731	1425.867 ± 276.140	1484.830 ± 253.943	< 0.001
NHR	0.072 ± 0.036	0.083 ± 0.040	0.088 ± 0.041	0.100 ± 0.048	< 0.001
LHR	0.037 ± 0.016	0.042 ± 0.017	0.046 ± 0.020	0.051 ± 0.020	< 0.001
MHR	0.008 ± 0.003	0.010 ± 0.004	0.011 ± 0.004	0.013 ± 0.006	< 0.001
PHR	4.224 ± 1.501	4.935 ± 1.804	5.314 ± 1.931	5.969 ± 2.313	< 0.001
PHQ-9	2.754 ± 3.079	3.440 ± 4.278	3.914 ± 4.662	4.174 ± 4.642	< 0.001
Race					< 0.001
Mexican American	84 (14.334%)	114 (19.757%)	108 (18.121%)	85 (14.480%)	
Other Hispanic	51 (8.703%)	69 (11.958%)	68 (11.409%)	60 (10.221%)	
Non-Hispanic White	182 (31.058%)	183 (31.716%)	177 (29.698%)	234 (39.864%)	
Non-Hispanic Black	108 (18.430%)	110 (19.064%)	158 (26.510%)	144 (24.532%)	
Other race (including multi-racial)	161 (27.474%)	101 (17.504%)	85 (14.262%)	64 (10.903%)	
Education					< 0.001
Below secondary school level	29 (4.949%)	42 (7.279%)	41 (6.879%)	20 (3.407%)	
High school students in grades 9 to 11	41 (6.997%)	52 (9.012%)	62 (10.403%)	72 (12.266%)	
High school graduates	80 (13.652%)	109 (18.891%)	132 (22.148%)	128 (21.806%)	
Holders of an associate's degree	196 (33.447%)	198 (34.315%)	224 (37.584%)	246 (41.908%)	
Holders of a postgraduate degree	240 (40.956%)	176 (30.503%)	137 (22.987%)	121 (20.613%)	
Naples prognostic score					0.341
Q1:0	78 (13.311%)	76 (13.172%)	79 (13.255%)	75 (12.777%)	
Q2:1–2	420 (71.672%)	418 (72.444%)	435 (72.987%)	402 (68.484%)	
Q3:3–4	88 (15.017%)	83 (14.385%)	82 (13.758%)	110 (18.739%)	
Depress					< 0.001
No	559 (95.392%)	526 (91.161%)	522 (87.584%)	508 (86.542%)	
Yes	27 (4.608%)	51 (8.839%)	74 (12.416%)	79 (13.458%)	
Obstructive sleep apnea					0.002
No	450 (76.792%)	439 (76.083%)	438 (73.490%)	398 (67.802%)	
Yes	136 (23.208%)	138 (23.917%)	158 (26.510%)	189 (32.198%)	
Hypertension					< 0.001
No	533 (91.267%)	497 (84.668%)	473 (80.855%)	453 (77.304%)	
Yes	51 (8.733%)	90 (15.332%)	112 (19.145%)	133 (22.696%)	
Diabetes					< 0.001
No	566 (96.918%)	552 (94.037%)	525 (89.744%)	530 (90.444%)	
Yes	11 (1.884%)	28 (4.770%)	49 (8.376%)	45 (7.679%)	
Borderline	7 (1.199%)	7 (1.193%)	11 (1.880%)	11 (1.877%)	
Sleep disorder					< 0.001
No	361 (61.604%)	306 (53.033%)	293 (49.161%)	249 (42.419%)	
Yes	225 (38.396%)	271 (46.967%)	303 (50.839%)	338 (57.581%)	

Results in table: mean + SD /N (%)

P-value: obtained by Kruskal–Wallis rank sum test for continuous variables, and Fisher’s exact probability test for counting variables with a theoretical number < 10

AGP: Q1: ≤ 0.622 g/L, Q2: > 0.622; ≤ 0.776, Q3: > 0.776; ≤ 0.94, Q4: > 0.948 (g/L)

*PIR* the ratio of income to poverty, *BMI* body mass index, *CRP* C-reactive protein, *NLR* neutrophil-to-lymphocyte ratio, *LMR* lymphocyte-to-monocyte ratio, *PHR* platelet to high-density lipoprotein ratio, *NHR* neutrophil to high-density lipoprotein ratio, *LHR* lymphocyte to high-density lipoprotein ratio, *MHR* monocyte to high-density lipoprotein ratio, *NPS* Naples Prognostic Score, *SII* immune-inflammatory index, *NPAR* neutrophil percentage-to-albumin ratio

### Development and validation of the comorbidity disease prediction model

**Fig. 2:**
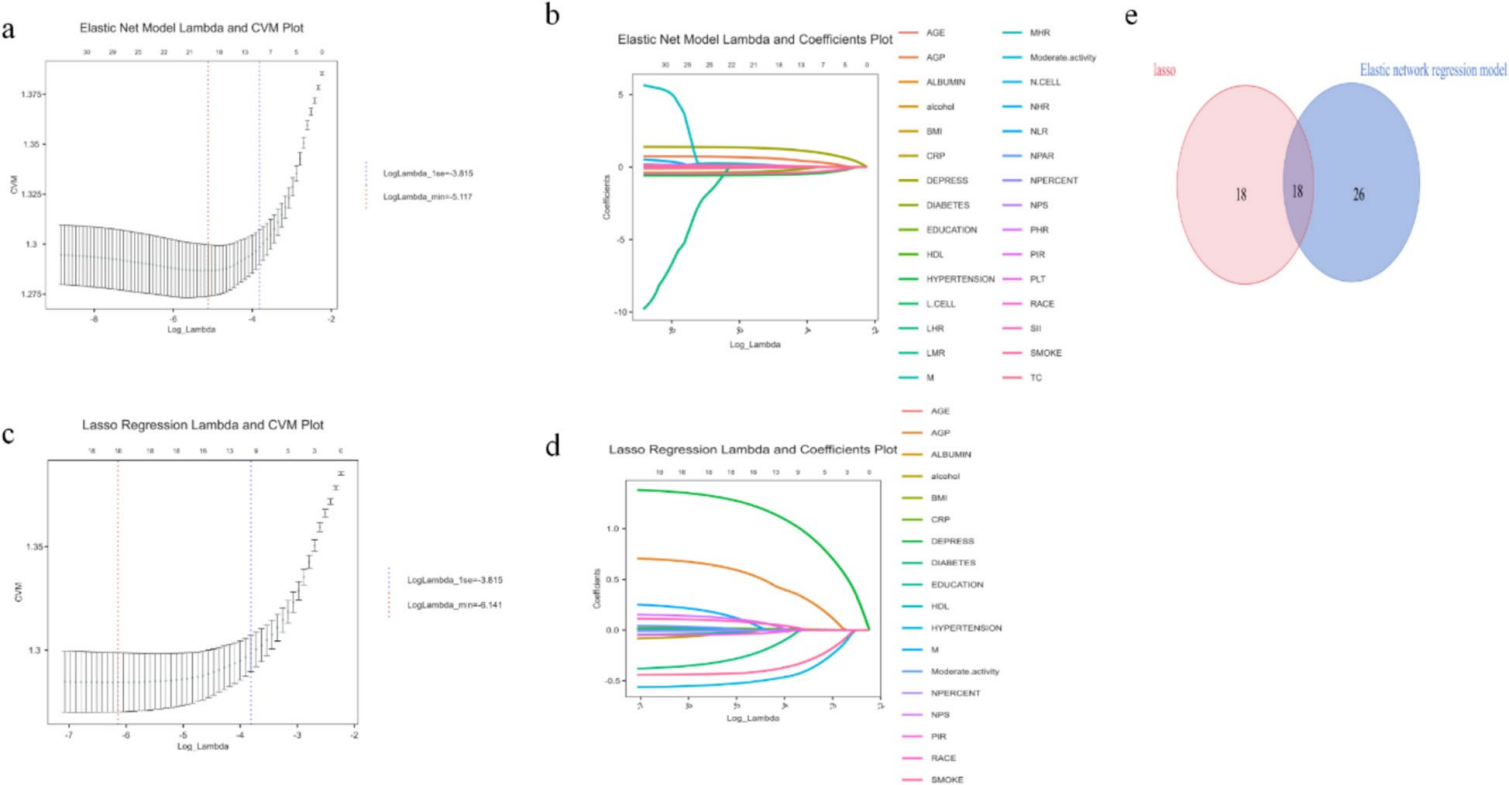
Feature selection process for variables included in the prediction model. **a**. Plots of the mean square error of lambda values for the elastic network model; **b**. plots of the coefficients of lambda values for the elastic network model; **c**. plots of the mean square error of lambda values for the lasso regression; **d**. plots of the coefficients of lambda values for the lasso regression, and **e**. Venn diagrams comparing the variables selected for the two different methods

**Fig. 3:**
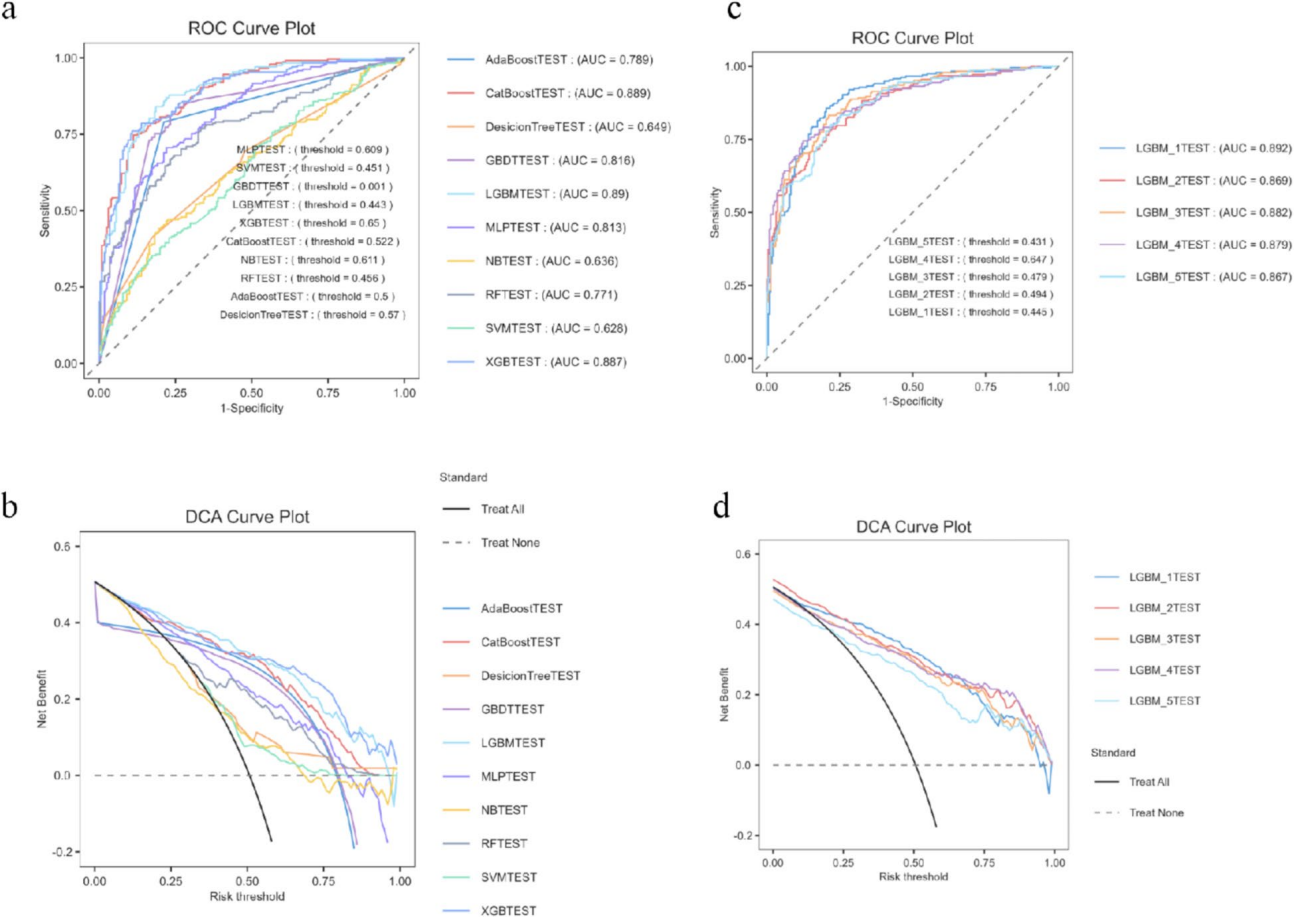
Predictive performance of the model in validation groups and cross-validation. **a**. ROC curves for the validation cohort; **b**. decision curve analysis (DCA) for the validation cohort; **c**. ROC curves for the quintuple-fold cross-validation; **d**. decision curve analysis (DCA) for the quintuple-fold cross-validation

Through the feature selection process, the elastic network regression model then selected 26 variables (Fig. 2b), while the LASSO regression identified 18 candidate biomarkers associated with sleep disorders (Fig. 2d). Cross-analysis identified 18 consensus predictors (Fig. 2e), including inflammatory markers (AGP, CRP, NPS, neutrophil percentage, monocytes, albumin), comorbidities (hypertension, diabetes, depression), lifestyle factors (smoking, alcohol consumption, physical activity), and demographic parameters (age, PIR, education level, ethnicity, BMI, HDL). PHR, NPAR, and NLR were excluded due to VIF > 10. Among the 11 machine learning models trained based on selected features, preliminary evaluation (Table 2) indicates that the LGBM model stands out: its classification accuracy (0.817), recall (0.786), and precision (0.842) form a favorable combination among all models, ensuring a certain case capture rate; the F1 score (0.813) confirms its balanced trade-off between recall and precision, placing it among the top performers overall. This model demonstrated excellent discriminative capability on the test set, achieving an AUC value of 0.890. The decision curve analysis indicates that within the moderate-to-high risk threshold range of 0.4–0.6, the LGBM model's net benefit approaches the optimal strategy, demonstrating stable clinical decision-making value. It also maintains good performance in the lower risk threshold range, highlighting its broad applicability. Fivefold cross-validation further confirms the LGBM model's high stability across different validation folds (Fig. 3).

**Table 2:** Indicators of 11 machine learning models in predicting sleep disorder

Model name	Accuracy	Prevalence	Recall	F1-Score	MCC	AUC	Precision	Specificity	FNR	FPR
DecisionTree	0.599	0.507	0.660	0.625	0.198	0.649	0.595	0.537	0.340	0.463
AdaBoost	0.789	0.507	0.790	0.792	0.578	0.789	0.793	0.788	0.210	0.212
RF	0.712	0.507	0.643	0.694	0.430	0.771	0.754	0.784	0.357	0.216
NB	0.595	0.507	0.538	0.574	0.193	0.636	0.615	0.654	0.462	0.346
CatBoost	0.802	0.507	0.761	0.796	0.606	0.889	0.834	0.844	0.239	0.156
XGB	0.800	0.507	0.798	0.802	0.599	0.887	0.805	0.801	0.202	0.199
LGBM	0.817	0.507	0.786	0.813	0.635	0.890	0.842	0.848	0.214	0.152
GBDT	0.774	0.507	0.756	0.773	0.549	0.816	0.789	0.792	0.244	0.208
SVM	0.586	0.507	0.416	0.505	0.189	0.628	0.643	0.762	0.584	0.238
MLP	0.723	0.507	0.769	0.738	0.446	0.813	0.709	0.675	0.231	0.325
Logistic	0.620	0.448	0.543	0.562	0.228	0.648	0.582	0.683	0.457	0.317
Mean-scores	0.711	0.502	0.678	0.697	0.423	0.765	0.724	0.743	0.322	0.257

### Importance of the inflammation indicators features interpreted by the SHAP value

**Fig. 4:**
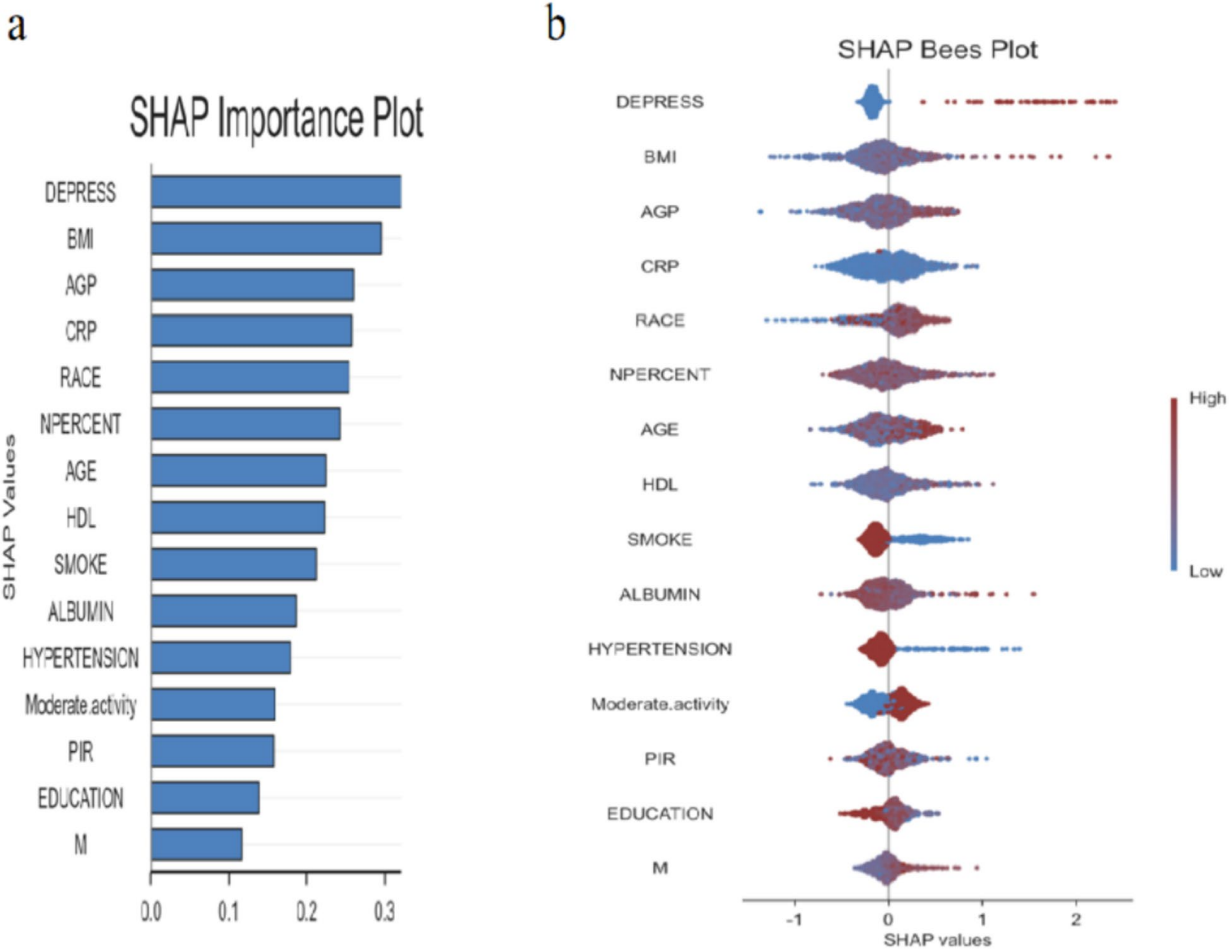
Shapley Additive exPlanations analysis of the model. *AGP* alpha-1-acid glycoprotein, *PIR* the ratio of income to poverty, *BMI* body mass index, *CRP* C-reactive protein, *PHR* platelet to high-density lipoprotein ratio, *NPS* Naples prognostic score, *NPAR* neutrophil percentage to albumin ratio

According to the SHAP importance plot (Fig. 4a), the top three variables are depression (DEPRESS, SHAP = 0.35), BMI (SHAP = 0.35), and AGP (SHAP = 0.35). The importance of all inflammation-related indicators is ranked as follows: AGP (SHAP = 0.35), CRP (SHAP = 0.35), HDL (SHAP = 0.35), albumin (SHAP = 0.18), and monocyte count (SHAP = 0.20). Specific SHAP values can be found in the supplementary file Table 1.

### Association between inflammation indicators and the odds of sleep disorder

**Fig. 5:**
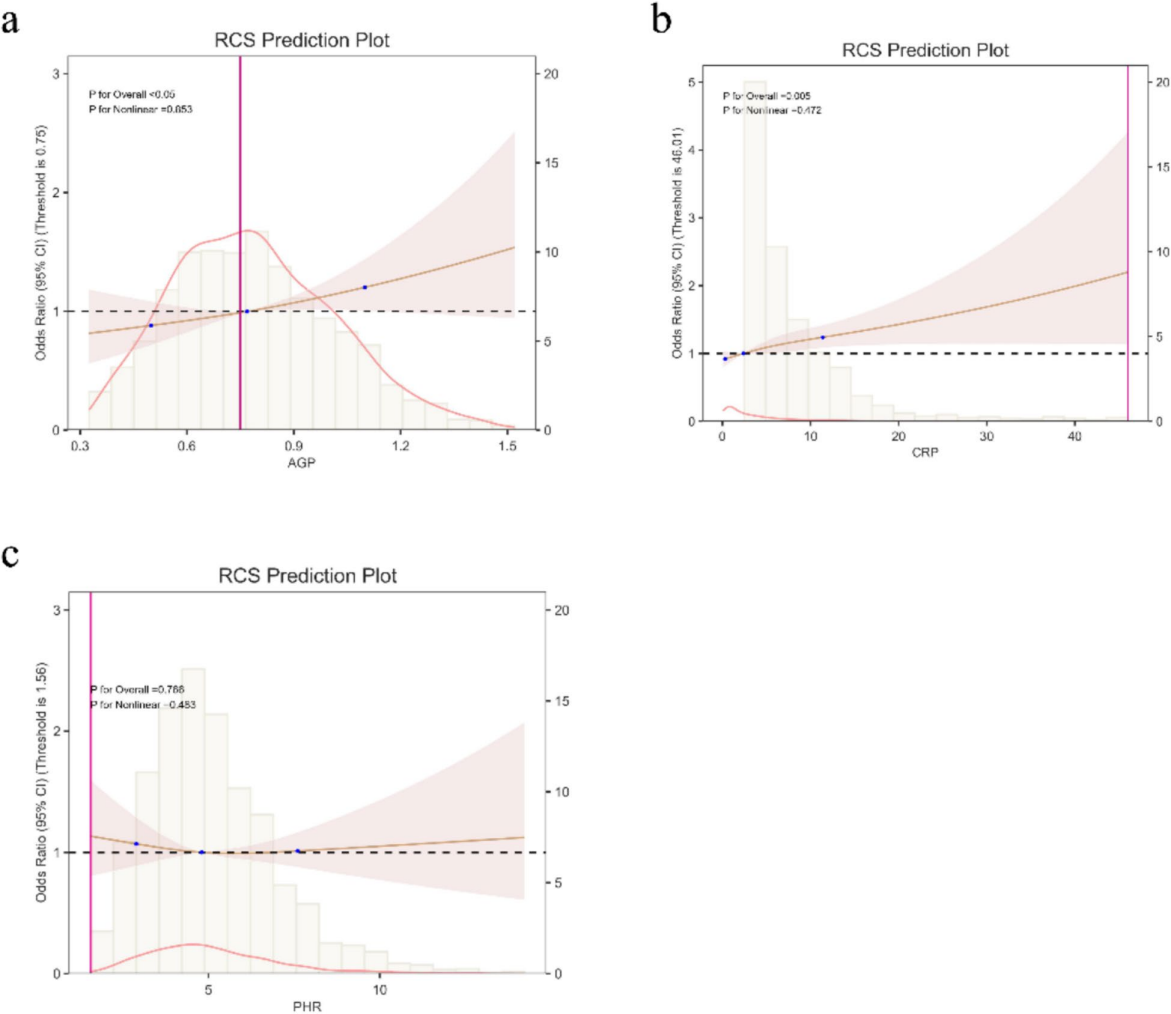
Restricted cubic spline plots between serum inflammatory markers and sleep disorders. **a**. Represents the restricted cubic spline plot between AGP and sleep disorders; **b**. represents the restricted cubic spline plot between CRP and sleep disorders; **c**. represents the restricted cubic spline plot between PHR and sleep disorders. The solid brown line represents the curve fit between the variables, and the red shading represents the 95% confidence interval of the fit

Table 3 data indicate that in all three models, AGP, NPS, CRP, and PHR were positively correlated with the probability of sleep disorders. Among these, when used as continuous variables and after adjusting for all confounding factors, AGP showed the strongest positive correlation with sleep disorders (OR = 1.583, 95% CI 1.029, 2.434), followed by NPS (OR = 1.119, 95% CI: 1.021, 1.228), and finally CRP (OR = 1.021, 95% CI 1.007, 1.035). PHR showed no statistical significance when treated as a continuous variable (*P* = 0.675). Furthermore, when these inflammatory markers were treated as categorical variables, CRP showed a negative correlation in the low-concentration group (Q2) (OR = 0.785, 95% CI 0.617, 0.998) and a positive correlation in the high-concentration group (Q4) (OR = 1.282, 95% CI 1.009, 1.629). AGP and NPS were statistically significant only in the highest concentration group, while PHR was statistically significant only in the low-concentration group (Q2). Figure 5a-c displays the restricted cubic sample plots of AGP, CRP, and PHR with sleep disorders, indicating no nonlinear relationship between AGP, CRP, and sleep disorders.

**Table 3:**
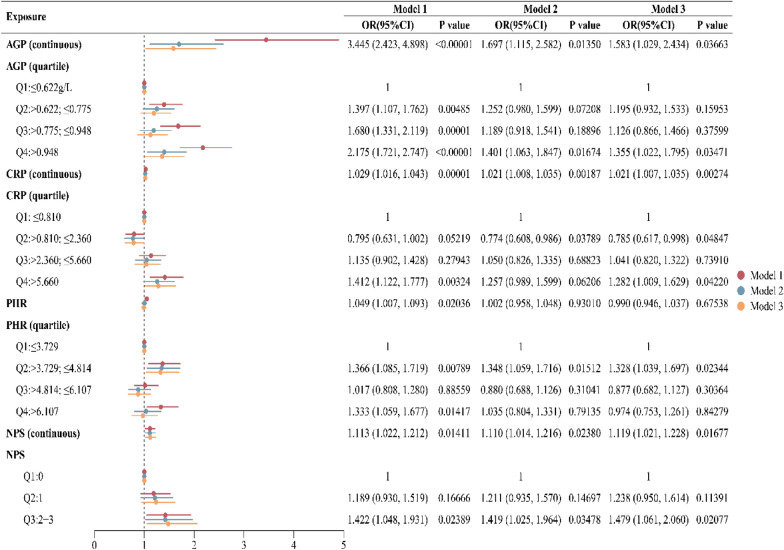
Associations between serum inflammation indicators and sleep disorders

In the sensitivity analysis, AGP, CRP, and PHR were converted from continuous variables to categorical variables (quartiles).

Model 1: No covariates were adjusted.

Model 2: age, BMI, family PIR, race, smoking, alcohol use, moderate activity, and education were adjusted.

Model 3: age, BMI, family PIR, race, education, smoking, alcohol use, moderate activity, depression, hypertension, and diabetes were adjusted.

*PIR* the ratio of income to poverty, *BMI* body mass index, *Q* quartile, *CRP* C-reactive protein, *NPS* Naples Prognostic Score, *PHR* platelet to high-density lipoprotein ratio.

### Predictive ability of individual inflammatory markers for sleep disorders

**Fig. 6:**
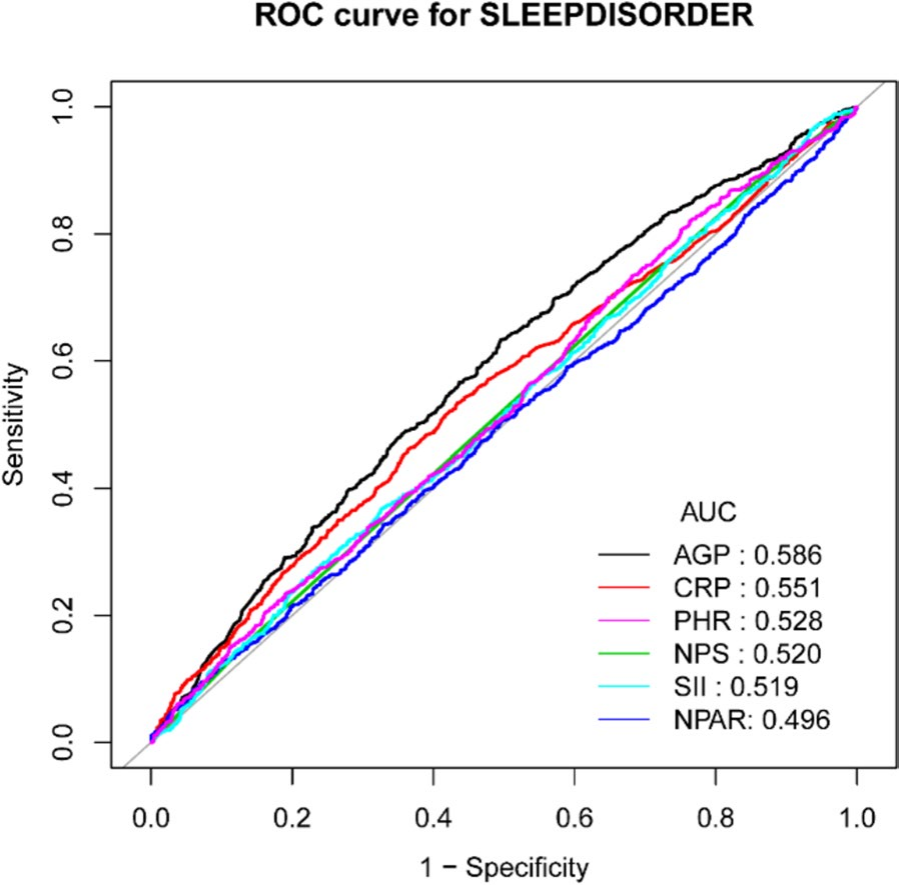
ROC curve between inflammation indicators and sleep disorder. *AGP* alpha-acid glycoprotein, *CRP* C-reactive protein, *PHR* platelet to high-density lipoprotein ratio, *NPS* Naples Prognostic Score, *SII* immune-inflammatory index, *NPAR* neutrophil percentage to albumin ratio

As shown in Fig. 6, AGP had the highest AUC value (AUC = 0.586). This suggests that AGP, as a single indicator, is a better predictor of sleep disorders than the commonly used inflammatory indicators mentioned above.

### Sensitivity analyses

**Fig. 7:**
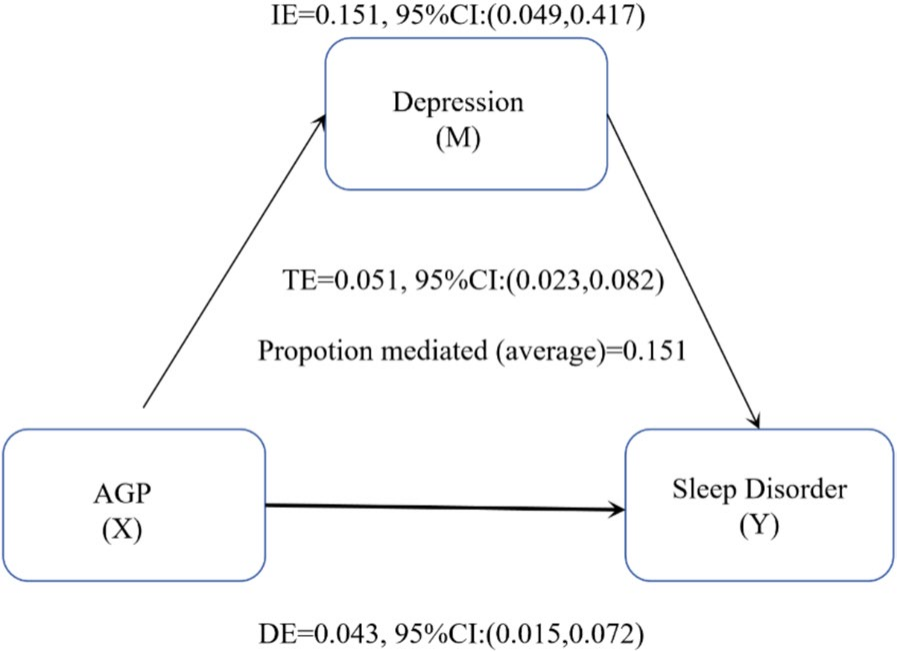
Mediation effects of depression on the associations of AGP and Sleep disorder. Age, BMI, family PIR, race, education, smoking, alcohol use, moderate activity, depression, hypertension, and diabetes were adjusted

**Fig. 8:**
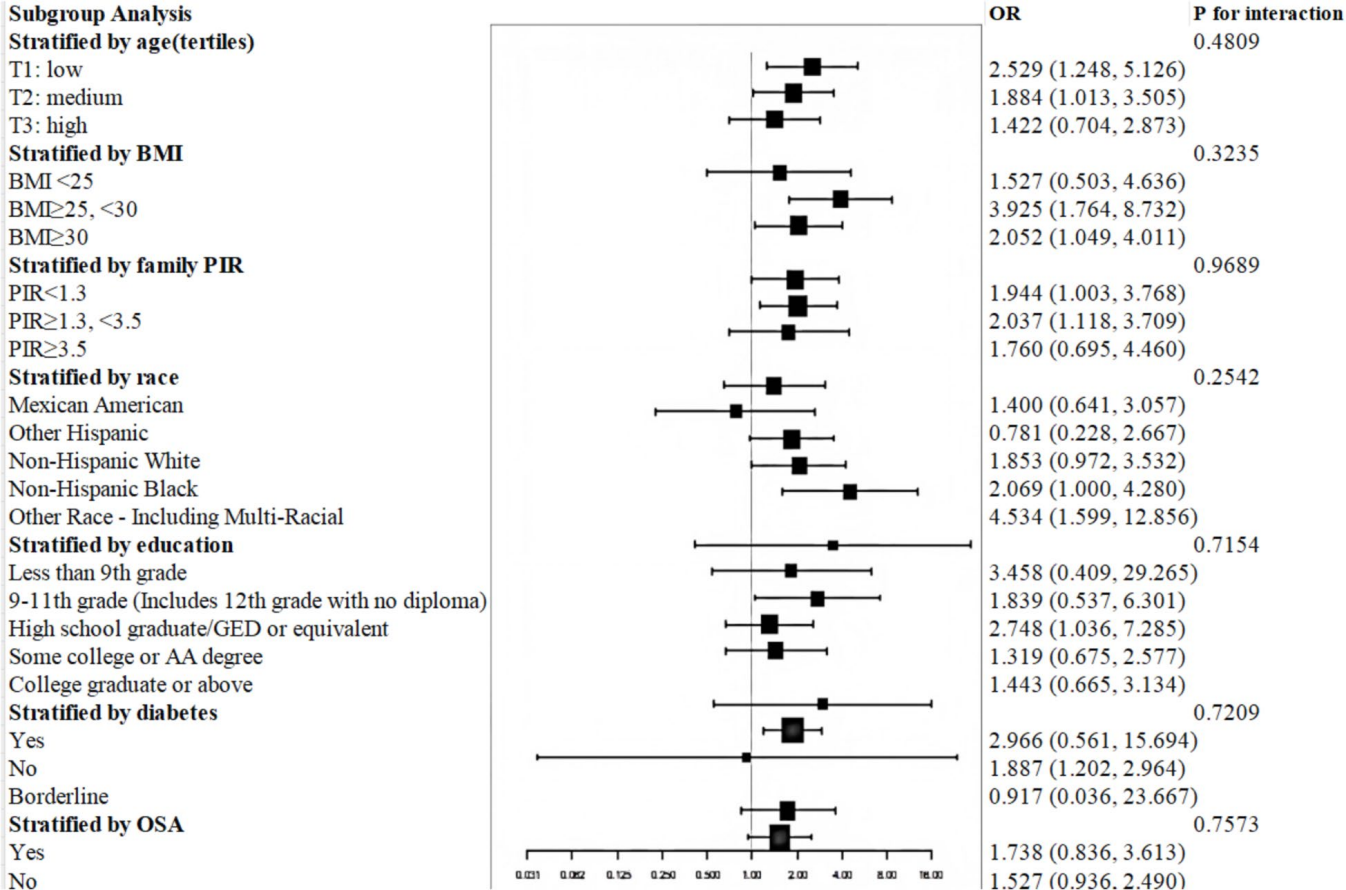
Subgroup analysis of the association between AGP and sleep disorder. Age, BMI, family PIR, race, education, smoking, alcohol use, moderate activity, depression, hypertension, and diabetes were adjusted

Mediation analyses (Fig. 7) showed that 15.1% (95% CI: 0.049, 0.417; P = 0.002) of the observed associations between AGP and sleep disorders were mediated by depression. Interaction tests for age, race, family PIR, education, BMI, OSA, and diabetes showed no significant effect modification (all P for interaction > 0.05), indicating that the association between serum AGP and sleep disorders did not vary substantially across these subgroups. Subgroup analyses revealed a consistent positive trend (OR > 1 in all strata), though statistical significance was not uniform. For example, in BMI categories, the association was significant in overweight individuals (OR = 3.925, 95%CI 1.764, 8.732) but not in normal weight (OR = 1.527, 95%CI 0.503,4.636) or obese subgroups (OR = 2.052, 95%CI 1.049,4.011). Similar patterns were observed in race and education subgroups (Fig. 8). These results suggest a general positive association between AGP and sleep disorders, with varying precision across populations. We assessed the sensitivity of the association between AGP and sleep disorders to unmeasured confounders using E-value analysis. The observed odds ratio was 1.705, requiring attenuation to 1.00 to eliminate this effect. This implies that unmeasured confounders must increase the risk of sleep disorders by at least 5.19-fold and increase the association risk with AGP by 2.05-fold.

## Discussion

This study investigated the association between multiple inflammatory indicators and sleep disorders in a cohort of 2,342 women aged 20–49 years. When analyzed as continuous variables, AGP, CRP, and NPS all demonstrated significant positive associations with sleep disorders. Notably, a nonlinear relationship was observed for CRP: lower concentrations (Q2: > 0.810 to ≤ 2.360 mg/L) were inversely associated with sleep disorders, whereas higher concentrations (Q4: > 5.660 mg/L) were positively associated. In contrast, PHR showed a positive association with sleep disturbance only in the Q2 subgroup (OR = 1.361, 95% CI 1.076, 1.723). Furthermore, mediation analysis revealed that depression partially mediated the relationship between AGP and sleep disorders, accounting for 15.1% of the total effect.

Several studies have confirmed that sleep disorders promote inflammation and that C-reactive protein (CRP) is higher in patients with sleep disorders [3], and levels of inflammatory substances such as CRP, IL-6 (interleukin 6), and TNF (tumor necrosis factor) decrease when insomnia resolves[2]. A study by Michael et al. summarized CRP levels in 34,000 sleep-disordered patients and IL-6 levels in more than 3000 sleep-disordered patients, confirming that CRP and IL-6 levels are increased in sleep-disordered patients [3]. In the findings of Michael et al., TNF levels did not seem to be associated with sleep disorders. However, since they only counted 672 subjects, there may be some bias. Interestingly, in this study, CRP exhibited a U-shaped relationship with sleep disorders. Specifically, it showed a negative correlation at low concentrations (Q2 group) and a positive correlation at high concentrations (Q4 group). This may be attributed to CRP's biphasic biological function, but whether it holds in patients with sleep disorders still requires extensive experimental verification. At baseline levels (typically < 3 mg/L), CRP is primarily produced by the liver under the influence of cytokine secretion, such as IL-6 [32]. It participates in innate immune regulation by recognizing and binding to membrane components of apoptotic and damaged cells, as well as certain pathogens, through its pentameric structure [33]. Via opsonization, it facilitates their clearance by macrophages, thereby maintaining homeostasis [34]. This constitutes a cleansing and repair function. When confronted with intense, persistent inflammatory stimuli (such as systemic inflammation resulting from chronic sleep disorders), CRP levels surge dramatically (typically > 5–10 mg/L). High CRP concentrations saturate and overwhelm regulatory mechanisms, strongly activating the classical complement pathway to form membrane attack complexes, causing tissue damage; promoting monocyte release of inflammatory mediators (TNF-α, IL-1β); and inducing endothelial dysfunction and oxidative stress [35, 36]. Furthermore, in this study, the negative correlation between PHR and sleep disorders was only significant at Q2, reflecting a nonlinear effect. Existing literature indicates that persistent sleep deprivation can lead to a significant reduction in high-density lipoprotein cholesterol (HDL-C) concentration [37]. Previous studies have further confirmed through gene expression analysis that lipid homeostasis is regulated by circadian clock genes associated with the sleep–wake cycle [38]. This physiological mechanism may partially explain our findings.

AGP (alpha-1-acid glycoprotein), a major acute-phase reactant [7], plays a significant role in the inflammatory response and is increasingly recognized for its dual roles in modulating systemic inflammation and neuroimmune interactions. Unlike C-reactive protein (CRP) or interleukin-6 (IL-6), which primarily reflect peripheral inflammatory status, AGP exhibits unique permeability across the blood–brain barrier due to its low molecular weight and lipophilic properties [39, 40]. This characteristic may enable AGP to directly interact with key components of the central nervous system, such as the hypothalamic–pituitary–adrenal axis [41]. Moreover, AGP significantly enhances the DNA-binding activity of the transcription factor Activator Protein-1 (AP-1). AP-1 is a key regulator of inflammatory genes such as IL-6 and TNF-α, and its activation suppresses the release of proinflammatory cytokines [42]. Proinflammatory cytokines (e.g., TNF-α, IL-1β) are established mediators of sleep fragmentation and non-restorative sleep [43, 44]; whereas, elevated AGP levels may exert anti-inflammatory effects by modulating microglia activation and migration [45, 46], thereby reducing neuroinflammation, this mechanism may occur at higher levels of inflammation. This is consistent with our observation that the dose–response relationship (Q3–Q4 significance) is consistent with this hypothesis, suggesting that AGP may have an effect on sleep architecture only when critical thresholds for CNS infiltration or immune activation are exceeded. However, more detailed and direct evidence is needed on whether it plays a role in the development of sleep disorders by suppressing inflammation. Additionally, among the biomarkers evaluated in this study, AGP demonstrated the highest discriminatory performance (AUC = 0.586). However, this value remains below the clinical diagnostic threshold requirement (AUC > 0.7), indicating insufficient accuracy for use as an independent diagnostic indicator. Therefore, the clinical utility of AGP when applied independently remains limited. This study aims to provide a new reference framework for developing relevant biomarkers through exploratory analysis. Additionally, regarding the clinical cutoff value for AGP, there is currently no universally agreed-upon threshold for future clinical use. Furthermore, the increase differs between acute and chronic inflammation. For predicting outcomes in adult sepsis patients, the optimal cutoff value is 1307 (μg/mL) [47]. In contrast, the mean value for chronic inflammation in hemodialysis patients was 1.01 (g/L) [48]. We conducted a threshold effect analysis for this scenario, which identified a breakpoint K of 1.03 (g/L). However, since our threshold effect analysis lacked statistical significance, it is difficult to provide compelling evidence to support its application in future clinical or screening scenarios. The limitations of AGP in practical applications align with certain academic viewpoints. Some scholars have noted that, compared to the widely used inflammatory markers IL-6 and CRP, AGP does not demonstrate superiority in predicting either short-term or long-term mortality risk [49]. The rationale behind this conclusion lies in the fact that during acute inflammatory responses in humans, CRP plasma concentrations can surge by a factor of a thousand [50], whereas AGP increases are relatively limited. Furthermore, the absence of a unified diagnostic threshold for AGP further constrains its clinical utility.

This study also revealed through mediation analysis that approximately 15.1% of the association between AGP and sleep disorders was mediated by depression. This may be related to inflammation disrupting the hypothalamic–pituitary–adrenal (HPA) axis [51]. First, AGP can activate TLR4 by binding to CD14, thereby increasing IL-6 release [52]. IL-6, widely reported as a key inflammatory mediator associated with depression, may lead to HPA axis dysfunction, altered synaptic neurotransmission, and reduced neurotrophic factors when elevated [51, 53]. This may validate the pathological pathway from inflammation to psychological stress and ultimately to sleep disorders [54], suggesting that patients with high AGP levels require combined anti-inflammatory and antidepressant interventions. However, current conclusions warrant cautious interpretation. On one hand, the association between IL-6 and depression exhibits heterogeneity, with some studies failing to observe a direct correlation [55]. This discrepancy may stem from variations in patients’ baseline inflammatory status, IL-6 gene polymorphisms, or sampling timepoints. On the other hand, whether AGP specifically drives IL-6 release from monocytes via the TLR4 pathway in patients with sleep disorders requires experimental validation.

In addition, patients with sleep disorders, especially insomnia, often experience chronic inflammation due to increased sympathetic nervous system activity, including anxiety and enhanced arousal [56]. This is associated with elevated levels of CRP, IL-6, and other inflammatory cytokines [57]. The role of AGP as a marker of inflammation in these processes requires more targeted studies to clarify whether AGP is an active participant in this complex relationship or merely a bystander due to the systemic inflammation associated with sleep disorders. Overall, while there is strong evidence supporting the involvement of inflammation in sleep disorders, the role of AGP in these processes remains less clear [58, 59]. Future studies are needed to better understand whether AGP contributes directly to the inflammatory pathways involved in sleep disorders or if its elevation is simply a reflection of the broader inflammatory response that characterizes these conditions.

The strength of the current study is the utilization of the NHANES database (large sample size and well-trained investigators), which improves the stability of the raw data and contributes greatly to the consistency of the results. Second, we searched the relevant literature; no studies have investigated the association between serum AGP and sleep disorders, and our study fills this gap. Additionally, we employed multivariate logistic regression and curve fitting to analyze the study population, adjusting for confounders. We also performed subgroup analyses to confirm that the association between serum AGP and sleep disorders was robust across all strata. Furthermore, we used E-value sensitivity analyses to quantify the potential effect of unmeasured confounders on the positive association between AGP and sleep disorders and found that unmeasured confounders were unlikely to explain the positive association between AGP and sleep disorders.

This study has several limitations. First, the cross-sectional design precludes definitive conclusions regarding causal relationships between serum AGP and sleep disorders. Second, although we adjusted for multiple covariates, residual confounding factors from unmeasured factors (such as hormone fluctuations and lifestyle factors) could not be completely ruled out. Critically, the modest sample size (*N* = 2342) reflects the deliberate restriction to a specific demographic—women aged 20–49 years—which prioritizes internal validity for this high-risk subgroup but limits generalizability to broader populations. Although our subgroup analysis of OSA yielded stable results, potential misclassification bias may still exist because our outcomes do not fully encompass clinical sleep disorders (e.g., insomnia and OSA), thereby affecting the stability and generalizability of the study findings. For individuals with insomnia, subjective sleep difficulties are often accompanied by sleep state misperception (SSM), resulting in questionnaire-reported subjective sleep difficulties covering a broader range than those measured by objective polysomnography [60]. Furthermore, since the NHANES questionnaire does not address sleep maintenance, insomnia, or early morning awakening, this may also lead to misinterpretation of outcomes. However, we believe that self-reported subjective sleep difficulties and sleep duration can partially reflect the characteristics of such patients. In addition, the reliance on self-reported measures for key variables—including health-related factors such as medical history and healthcare utilization—introduces methodological limitations that may compromise the validity of the findings. Future longitudinal studies with larger, age-diverse cohorts are warranted to validate these findings. Additionally, in subgroup analyses, we observed notably wide confidence intervals for certain subgroups—such as the BMI < 25 group, specific racial groups like “Other races—including multiracial”, educational attainment “below 9th grade”, and the diabetes “borderline” group. This indicates that our study lacked sufficient statistical power in these specific populations due to limited sample sizes, resulting in high uncertainty in effect estimates. Therefore, interpretation of results for these subgroups should be approached with particular caution. Point estimates should not be interpreted as reliable evidence, but rather regarded as exploratory findings that require validation in future, larger-scale studies. The model that performed best in this study was LGBM. Although it possesses a robust anti-overfitting mechanism, and we employed cross-validation to mitigate overfitting risks, overfitting could not be entirely avoided. This issue warrants continued attention in future research.

## Conclusion

In conclusion, AGP, CRP, and NPS all showed significant positive correlations with sleep disorders when treated as continuous variables. However, stratified analysis revealed threshold effects in these associations: AGP and NPS maintained positive correlations only in the highest concentration group (Q4), while CRP exhibited a unique U-shaped relationship (negative correlation in the low-concentration Q2 group and positive correlation in the high-concentration Q4 group). PHR showed a positive correlation only in the Q2 group. Depression partially mediated the positive association between AGP and sleep disorders (mediation ratio: 15.1%). Although AGP exhibited the highest SHAP value among inflammatory markers in the machine learning model (LGBM), its independent ability to distinguish sleep disorders remained insufficient, suggesting that multidimensional indicators should be integrated to optimize clinical predictive efficacy.

## Supplementary Information

Additional file 1: Table 1: SHAP value results

## Data Availability

All data files are available from the NHANES (www.cdc.gov/nchs/nhanes/).

## Abbreviations

AGP: Alpha-acid glycoprotein
ORM: Orosomucoid
IL-1 β: Interleukin-1 β
IFN: Type I and type II interferons
TNF α: Tumor necrosis factor-α
IL-6: Interleukin-6
PIR: Income-to-poverty ratio
CRP: C-reactive protein levels
SII: Immune-inflammatory index
NLR: Neutrophil-to-lymphocyte ratio
LMR: Lymphocyte-to-monocyte ratio
PHR: Platelet to high-density lipoprotein ratio
NHR: Neutrophil to high-density lipoprotein ratio
LHR: Lymphocyte to high-density lipoprotein ratio
MHR: Monocyte to high-density lipoprotein ratio
NPS: Naples Prognostic Score
NPAR: Neutrophil percentage to albumin ratio
