# Supplementary material for: Assessing the association between multiple indicators of inflammation and sleep disorders in young and middle-aged women: insights from traditional and machine learning approaches
Source: Eur J Med Res. 2025 Oct 15;30:972. doi: 10.1186/s40001-025-03203-0 (PMC12522555; doi:10.1186/s40001-025-03203-0)
Supplement: Supplementary file 1 — Additional file 1: Table 1: SHAP value results [file 40001_2025_3203_MOESM1_ESM.docx]

**Specific Measurement Methods for AGP**

AGP is an acute-phase protein in plasma that is used to assess the inflammatory response. Through immunoagglutination, the anti-α1-Acid Glycoprotein antibody reacts with the antigen in the sample to generate an antigen/antibody complex, which is then used to detect AGP using the Tina-quant Roche AAGP2 assay. A turbidimetric assay is subsequently used to quantify this agglutination (see the package insert for AAGP2 Tina-quant α1-Acid Glycoprotein Gen. 2). Using a multi-role quality control process, two levels of Roche QC mixes or three levels of serum QC mixes that were made in-house were examined in duplicate and their validity assessed using pre-established means and control limits. Following the completion of all laboratory analyses, data collection took place[1].

**The calculation method for composite indicators**

The construction of the Naples prognostic score (NPS) was based on the criteria proposed by Galizia et al. and was accomplished by integrating four parameters: serum albumin, total cholesterol (TC), neutrophil-lymphocyte ratio (NLR), and lymphocyte-monocyte ratio (LMR)[2]. The threshold values were defined by MaxStat statistical analysis: when the serum albumin concentration ≥40 g/L, TC level >180 mg/dL, NLR value <2.96, or LMR >4.44, the corresponding parameter was assigned a score of 0; conversely a score of 1 was assigned if serum albumin <40 g/L, TC ≤180 mg/dL, NLR ≥2.96, or LMR ≤4.44[2]. The final NPS value was the sum of the four index scores, and the subjects were divided into three prognostic groups - Group 1 (0 points), Group 2 (1-2 points), and Group 3 (3-4 points) as the basis for disease risk stratification. The remaining inflammatory indices were calculated using the same biological samples: systemic immune-inflammatory index (SII) was determined from the product of platelet count and neutrophil count divided by lymphocyte count (SII = platelets × neutrophils/lymphocytes); neutrophil percentage-albumin ratio (NPAR) was calculated as the value of the percentage of neutrophils to the total number of leukocytes multiplied by 100 and then divided by serum albumin concentration (g/dL) to arrive at (NPAR = neutrophils% × 100/albumin)[3]. For the lipoprotein-immunocyte series ratios (MHR, LHR, NHR, PHR), the NPAR was directly based on monocyte count (MON), lymphocyte count (LYM), neutrophil count (NEU), platelet count (PLT), and high-density lipoprotein cholesterol (HDL-C) data provided by the NHANES complete blood count database, and was obtained through the Lymphocyte/ HDL-C (LHR), monocyte/HDL-C (MHR), neutrophil/HDL-C (NHR), and platelet/HDL-C (PHR formulas were completed.[4]

Supplementary Table 1: SHAP value results

|  | mean | median |
| --- | --- | --- |
| AGE | -0.0117 | -0.0204 |
| RACE | 0.0113 | 0.0807 |
| EDUCATION | 0.0235 | 0.0427 |
| PIR | 0.0207 | -0.0100 |
| BMI | -0.0425 | -0.0675 |
| HYPERTENSION | 0.0091 | -0.0721 |
| DIABETES | 0.0068 | -0.0476 |
| Moderate activity | -0.0030 | 0.0121 |
| alcohol | 0.0043 | 0.0029 |
| SMOKE | 0.0262 | -0.0897 |
| M | -0.0074 | -0.0217 |
| NPERCENT | 0.0058 | -0.0298 |
| ALBUMIN | 0.0004 | -0.0348 |
| HDL | 0.0103 | -0.0283 |
| AGP | -0.0556 | -0.0624 |
| DEPRESS | 0.0141 | -0.1651 |
| CRP | -0.0085 | -0.0307 |
| NPS | -0.0016 | 0.0157 |

**References**

1. Caudill SP, Schleicher RL, Pirkle JL: **Multi-rule quality control for the age-related eye disease study**. *Stat Med* 2008, **27**(20):4094-4106.

2. Galizia G, Lieto E, Auricchio A, Cardella F, Mabilia A, Podzemny V, Castellano P, Orditura M, Napolitano V: **Naples Prognostic Score, Based on Nutritional and Inflammatory Status, is an Independent Predictor of Long-term Outcome in Patients Undergoing Surgery for Colorectal Cancer**. *Dis Colon Rectum* 2017, **60**(12):1273-1284.

3. Zhang K, Ma X, Zhou X, Qiu G, Zhang C: **Machine learning based association between inflammation indicators (NLR, PLR, NPAR, SII, SIRI, and AISI) and all-cause mortality in arthritis patients with hypertension: NHANES 1999-2018**. *Front Public Health* 2025, **13**:1559603.

4. Zhao J, Zheng Q, Ying Y, Luo S, Liu N, Wang L, Xu T, Jiang A, Pan Y, Zhang D: **Association between high-density lipoprotein-related inflammation index and periodontitis: insights from NHANES 2009-2014**. *Lipids Health Dis* 2024, **23**(1):321.
